# Effect of early dietary energy restriction and phosphorus level on subsequent growth performance, intestinal phosphate transport, and AMPK activity in young broilers

**DOI:** 10.1371/journal.pone.0186828

**Published:** 2017-12-14

**Authors:** Zhiqiang Miao, Guixian Zhang, Junzhen Zhang, Jianhui Li, Yu Yang

**Affiliations:** College of Animal Science and Veterinary Medicine, Shanxi Agricultural University, Shanxi, China; Georgia Regents University, UNITED STATES

## Abstract

We aimed to determine the effect of low dietary energy on intestinal phosphate transport and the possible underlying mechanism to explain the long-term effects of early dietary energy restriction and non-phytate phosphorus (NPP). A 2 × 3 factorial experiment, consisting of 2 energy levels and 3 NPP levels, was conducted. Broiler growth performance, intestinal morphology in 0–21 days and 22–35 days, type IIb sodium-phosphate co-transporter (NaP_i_-IIb) mRNA expression, adenylate purine concentrations in the duodenum, and phosphorylated adenosine monophosphate-activated protein kinase (AMPK-α) activity in 0–21 days were determined. The following results were obtained. (1) Low dietary energy (LE) induced a high feed conversion ratio (FCR) and significantly decreased body weight gain in young broilers, but LE induced significantly higher compensatory growth in low NPP (LP) groups than in the high or medium NPP groups (HP and MP). (2) LE decreased the villus height (VH) in the intestine, and LE-HP resulted in the lowest crypt depth (CD) and the highest VH:CD ratio in the initial phase. However, in the later period, the LE-LP group showed an increased VH:CD ratio and decreased CD in the intestine. (3) LE increased ATP synthesis and decreased AMP:ATP ratio in the duodenal mucosa of chickens in 0–21 days, and LP diet increased ATP synthesis and adenylate energy charges but decreased AMP production and AMP:ATP ratio. (4) LE led to weaker AMPK phosphorylation, higher mTOR phosphorylation, and higher NaP_i_-IIb mRNA expression. Thus, LE and LP in the early growth phase had significant compensatory and interactive effect on later growth and intestinal development in broilers. The effect might be relevant to energy status that LE leads to weaker AMPK phosphorylation, causing a lower inhibitory action toward mTOR phosphorylation. This series of events stimulates NaP_i_-IIb mRNA expression. Our findings provide a theoretical basis and a new perspective on intestinal phosphate transport regulation, with potential applications in broiler production.

## Introduction

Since 1957, selective breeding has significantly increased growth in broiler chickens [1], leading to an increase in leg weakness and incidence of metabolic diseases, such as ascites, sudden death syndrome, and fat deposition [2–5]. Quantitative and qualitative feed restrictions in the early growth period were previously used to limit the growth rate and prevent these metabolic diseases [6]. These measures can improve meat quality and enhance resistance to diseases [7]. Early growth conditions play a decisive role in later growth, and early nutritional status can induce metabolic programming, resulting in long-term effects on later growth and meat quality [8].

Dietary energy and phosphorus are 2 critical components of animal diets, which affect the growth rate directly. An increase in the dietary energy level improves the growth rate [9] and increases the phosphorus requirements of chickens [10, 11]. High-energy diets may also increase lipid accumulation in broilers [12]. Feed restriction has been shown to have a positive effect on the intestinal ecosystem, promote the development of the digestive system of broilers [13, 14], and improve the intestinal structure in chickens [15, 16]. However, to the best of our knowledge, no study has evaluated the interactive effect of dietary energy and phosphorus, particularly during the early growth period, on later growth performance.

Inorganic phosphate (P_i_) is essential for bone mineralization and several other biological processes. The rate of intestinal P_i_ absorption is a major determinant of P_i_ homeostasis. The type IIb sodium-phosphate cotransporter (NaP_i_-IIb) is a critical transport protein for phosphate uptake in the small intestine, particularly in the duodenum [17]. Many factors regulate the rate of P_i_ absorption by modulating the expression of NaP_i_-IIb in the intestine, such as age, dietary calcium:phosphorus ratio, 1,25(OH)_2_D_3_, P_i_, parathyroid hormone, epidermal growth factor, and dietary nutrients [18–23].

The dietary energy level affects the growth rate of chickens and their phosphorus requirements, which might be correlated with intestinal absorption [9]. Chronic caloric restriction significantly reduces NaP_i_-IIb expression in aged mice, and this intestinal phosphate transporter is known to respond to energy levels in enterocytes [24]. The phenotypic expression of feed efficiency in broilers is related with the functioning of intestinal mitochondria [25]. However, to the best of our knowledge, the effect of dietary energy on intestinal phosphate transporter expression and the underlying regulatory mechanism in young broilers have not been reported until date.

AMP-activated protein kinase (AMPK), which is a sensor of peripheral energy balance, can be phosphorylated and activated by metabolic changes and an increase in the AMP:ATP ratio [26]. Once activated, AMPK switches off ATP-consuming biosynthetic pathways and switches on ATP-generating metabolic pathways. Feeding behavior has been demonstrated to be regulated by AMPK phosphorylation in the hypothalamus [27]. In addition, NaP_i_-IIb gene expression in the intestine is linked to the metabolic state of cells through AMPK activity, and AMPK has an inhibitory effect on NaP_i_-IIb in rats [28]. However, no data are available on the effect of dietary energy on AMPK during the process of phosphate absorption in broilers.

In the present study, we performed a 2 × 3 factorial experiment to investigate the interactive effect of energy and phosphorus status in early diets on subsequent growth performance, intestinal development, expression of intestinal NaPi-IIb, energy status, and AMPK activity. Our aim was to determine the effect of low dietary energy on intestinal phosphate transport and the possible underlying mechanism to explain the long-term effects of early dietary energy restriction and non-phytate phosphorus (NPP), which might provide a new perspective on intestinal phosphate transport, with potential applications in broiler production.

## Materials and methods

### Animals and diets

The present study was performed in accordance with the Guidelines for Experimental Animal Welfare of the Ministry of Science and Technology of China (Beijing, P. R. China), after approval. A total of 432 Arbor Acres Plus broilers (1 day old) purchased from a local hatchery were selected and randomly allocated to 36 pens, with 12 chickens in each pen.

Six diets were prepared for a 2 × 3 factorial experiment, consisting of 2 levels of energy and 3 levels of NPP (Table 1). The 2 different dietary energy levels were set at 12.12 MJ/kg (LE) and 13.37 MJ/kg (NE). The NPP levels in the diet were 0.23%, 0.45%, and 0.68%. Each of the 6 diets was fed to 6 replicate pens. Crude protein and essential amino acid content was maintained at the same level in all the treatments using an ideal amino acid pattern. These values were based on the recommendations of the National Research Council in 1994. After the broilers turned 21 days old, they were fed with the same basal diet until the age of 35 days. The dietary composition and nutrient content are shown in Table 1.

**Table 1:** Composition of diets and nutrient levels in broilers.

	0–21 days		22–35 days
	Low energy (LE)	Medium energy (ME)	
**Ingredients, %**			
Corn	48.49	41.44	53.18
Soybean meal	43.32	44.72	35.59
Soybean oil	3.72	9.36	7.60
Various^1^	3.45	3.46	2.65
DL-methionine	0.16	0.16	0.12
Salt	0.30	0.30	0.3
Trace mineral premix^2^	0.20	0.20	0.20
Vitamin premix^3^	0.03	0.03	0.03
Choline chloride 50%	0.30	0.30	0.30
Antioxidant	0.03	0.03	0.03
Total	100	100	100
**Nutrient composition**			
ME,MJ/kg	12.12	13.37	13.37
CP^5^,%	23.00	23.00	20.00
Calcium,%	1.00	1.00	0.90
Non-phytate phosphorus^4^,%	0.23	0.23	0.35
Lysine,%	1.18	1.20	1.07
Methionine,%	0.52	0.52	0.40
Tryptophan,%	0.33	0.33	0.23
Threonine,%	0.96	0.97	0.76

^1^Variable amounts of dicalcium phosphate, limestone, or maifanite.

^2^Nutrients per kilogram of diet: Cu (from CuSO_4_·5H_2_O), 16 mg; Fe (from FeSO_4_·7H_2_O), 80 mg; Zn (from ZnSO_4_·7H_2_O), 110 mg; Mn (from MnSO_4_·H_2_O), 120 mg; I (from Ca(IO_3_)_2_·H_2_O), 1.5 mg; Co (from CoCl_2_·6H_2_O), 0.5 mg; Se (from organic selenium), 0.3 mg.

^3^Nutrients per kilogram of diet: vitamin A, 12,500 IU; vitamin D_3_, 3,000 IU; vitamin E, 25 mg; vitamin K_3_, 2.5 mg; thiamin, 2.5 mg; riboflavin, 8 mg; vitamin B_12_, 0.025 mg; folic acid, 1.25 mg; niacin, 37.5 mg; pantothenic acid, 12.5 mg; biotin, 0.125 mg.

^4^Dietary non-phytate phosphorus (NPP) levels in the diet of 0–21 days were 0.23, 0.45, and 0.68%. The analyzed levels of NPP were 0.23, 0.46, and 0.67%; 0.23, 0.45, and 0.70% in each energy level. NPP level in the diet of 22–35 days was 0.36%.

^5^Crude protein (CP) content of corn is 8.7%, CP content of soybean meal is 43%.

The chickens were reared under controlled environments. The temperature was initially maintained at 34°C when the broilers were 1–3 days old, followed by a gradual decrease to room temperature (24°C). The light regimen was 23L:1D (light: dark). All the birds were provided mashed feed and tap water *ad libitum*.

### Sample collection

At the age of 21 and 35 days, the body weight of each broiler and the weight of the feed remaining in the trough were measured 4 h after feed withdrawal. Body weight gain (BWG), feed intake (FI), and feed conversion ratio (FCR) from days 1–21 and 22–35 and the final body weight of 35-day-old broilers were calculated.

After the birds were weighed, 1 bird from each pen, weighing closest to the mean body weight for each treatment, was killed by cervical dislocation. One-centimeter-long sections of duodenal tissues from 21- and 35-day-old broilers were carefully obtained and immediately fixed in 10% formaldehyde phosphate buffer for microscopic assessment of mucosal morphology. In addition, 10-cm segments of duodenal tissues of 21-day-old broilers were cut longitudinally, and the contents were flushed with ice-cold PBS. Mucosal samples were rapidly collected by scraping with a sterile glass microscope slide, immediately frozen in liquid nitrogen, and stored at -80°C until analysis.

### Morphological analysis of the small intestine

Intestinal samples were embedded in paraffin, and 5-μm-thick cross-sections were cut and mounted on polylysine-coated slides [29]. Thereafter, the slides were stained with hematoxylin and eosin for histological evaluation. Villus height (VH) and crypt depth (CD) were measured on the stained sections (4× objective) using a light microscope fitted with an image analyzer (Image Pro Plus, Media Cybernetics, Buckinghamshire, UK). VH was measured from the villus tip to the villus-crypt junction, and CD was measured from this junction to the base of the crypt. At least 15 well-oriented and intact villi and their associated crypts were measured in each slide.

### Determination of mucosal ATP, ADP, and AMP

Frozen mucosal samples (100–200 mg) were homogenized with 2 mL pre-cooled 1.5 mmol/L sodium fluoride-perchloric acid in an ice-bath [30]. The homogenates were centrifuged at 3000 ×*g* for 10 min at 4°C. One milliliter of the supernatant was neutralized with 0.4 mL of 2 M potassium carbonate on ice, and the solution was centrifuged at 3000 ×*g* for 5 min at 4°C. The ATP, ADP, and AMP content was analyzed according to the method of Hou *et al*. [31] using high-performance liquid chromatography. Total adenine nucleotide (TAN) and adenylate energy charges (AEC) were calculated according to the following equation [32]:

TAN=ATP+ADP+AMP,

AEC=(ATP+0.5ADP)/TAN.

### Total RNA extraction, reverse transcription, and real-time PCR

Total RNA was extracted from the duodenal mucosa of the 21-day-old broilers using the SV Total RNA Isolation System Kit (Z3100; Promega, Madison, WI, USA), according to the manufacturer’s instructions. The RNA was re-suspended in diethyl pyrocarbonate-treated water. The concentration and quality of the RNA were determined by measuring the absorbance at 260 nm and agarose gel electrophoresis, respectively.

After extraction, 1.0 μg total RNA was used as the template for synthesizing single-stranded cDNA with avian myeloblastosis virus reverse transcriptase (Promega, Madison, WI, USA) using a 15-mer oligo (dT) primer in the presence of recombinant RNasin ribonuclease inhibitor (A3500; Promega, Madison, WI, USA).

Real-time PCR of NaP_i_-IIb was performed with β-actin as the internal control. The primers and amplicon sizes are presented in Table 2. Real-time PCR was conducted in an ABI 7500 Fluorescent Quantitative PCR system (Applied Biosystems, Bedford, MA) using the RealSuper mixture with Rox (CW0767; CWbio Company, Sandringham, UK). The PCR conditions were as follows: 95°C for 4 min, followed by 40 cycles of 95°C for 15 s and 60°C for 60 s, and 60–95°C for melting curve analysis. Each gene was amplified in triplicate. Standard curves were analyzed simultaneously to determine the efficiency of amplification. The results were expressed as the ratio of the target gene mRNA to β-actin mRNA, and the differences in gene expression were determined using the cycle threshold method [22].

**Table 2:** Oligonucleotide PCR primers.

Gene	GenBank accession	Orientation	Primer sequence (5ʹ to 3ʹ)	Predicted size, bp
NaPi-IIb	NM_204474.1	Forward	CTTTTACTTGGCTGGCTGGAT	148
		Reverse	AGGGTGAGG**G**GATAAGAACG	
β-Actin	NM_205518.1	Forward	AACACCCACACCCCTGTGAT	100
		Reverse	TGAGTCAAGCGCCAAAAGAA	

### Western blot analysis

The duodenal brush-border membrane vesicles were homogenized and centrifuged at 10,000 ×*g* for 5 min at 4°C. The resulting supernatants were stored at -80°C until further use. Protein concentration was determined by the Bradford assay. The samples of brush-border membrane vesicles were placed in Laemmli buffer (Sigma-Aldrich, St. Louis, MO, USA) and boiled for 5 min to induce protein denaturation. Thereafter, 50 mg of brush-border membrane vesicle proteins were loaded onto each lane and electrophoresed on a 4% polyacrylamide gel. The proteins were subsequently transferred onto a polyvinylidene difluoride membrane for 2 h, followed by probing the polyvinylidene difluoride membrane for the presence of total and phosphorylated AMPK-α, and total and phosphorylated mammalian target of rapamycin (mTOR and p-mTOR) (AMPK-α antibody: no. 2532; phosphorylated AMPK-α antibody: no. 2531; mTOR: no. 2983; p-mTOR: no. 2971; Cell Signaling Technology, Inc., Beverly, MA, USA) by incubation with a primary antibody, diluted to 1:1000, for at least 1 h. The membrane was washed in Tris-buffered saline Tween-20 and incubated with a secondary antibody conjugated with horseradish peroxidase (1:5000; Bio-Rad, Hercules, CA, USA). The immunoblots were visualized on an X-ray film through chemiluminescence (Pierce Protein Research Products, Rockford, IL, USA), and optical density-calibrated images were analyzed using AlphaEase stand-alone software (Alpha Innotech, Santa Clara, CA, USA) [33]. Both the AMPK and p-AMPK band density were quantified, and the ratio of p-AMPK: total AMPK was calculated. The ratio of p-mTOR: total mTOR was also calculated after quantifying the mTOR and p-mTOR band density.

### Statistical analyses

The results of the 2 × 3 factorial experiments were analyzed with a general linear model by using SPSS ver. 17.0 (IBM-SPSS, Inc., Chicago, IL, USA) to estimate the effects of dietary energy, phosphorous, and their interaction [22]. Replicate means were used as the experimental unit in this analysis. The two-way analysis of variance output of the model was used to detect the significance of the explanatory variables. When a treatment was significant at *p* < 0.05, the differences between the means were assessed using Tukey’s honest significant difference multiple range analysis. Prior to analysis, the homogeneity of variance was examined, and the normality of data was verified.

## Results

### Early energy and phosphorus levels on subsequent growth performance and intestinal development

#### Growth performance

In the early dietary regimen, weight gain and feed intake were significantly affected by dietary energy level, NPP, and their interaction (p < 0.01) (Table 3). In the medium (MP) and high NPP (HP) dietary groups, LE showed no difference in FI but demonstrated significantly decreased body weight gain (BWG) compared with NE. In the low NPP (LP) group, LE increased the FI but showed no difference in BWG compared with NE. FCR was significantly affected by dietary energy level (p < 0.001) and NPP (p < 0.05). LE and LP both significantly increased FCR.

**Table 3:** The influence of different dietary energy and NPP levels on growth performance of broilers (n = 6).

		0–21 days			21–35 days			Final body weight, g
Dietary energy	NPP^1^	BWG, g^2^	FI, g^3^	FCR^4^	BWG, g	FI, g	FCR	
**Interaction effects**								
LE	LP	579.12^A^	737.78^B^	1.36	1,142^b^	1,738^b^	1.52	1,731^A^
	NP	785.13^B^	988.98^C^	1.33	1,230^b^^c^	1,981^c^	1.61	2,025^B^
	HP	785.58^B^	965.78^C^	1.29	1,255^c^	2,025^c^	1.61	2,051^B^^C^
NE	LP	560.15^A^	623.47^A^	1.21	1,032^a^	1,567^a^	1.52	1,598^A^
	NP	846.53^C^	947.36^C^	1.17	1,277^c^	2,062^c^	1.59	2,140^B^^C^
	HP	872.36^C^	991.14^C^	1.19	1,266^c^	2,052^c^	1.56	2,199^C^
SEM^5^		4.52	5.76	0.006	12.17	17.15	0.008	14.31
Main effects*								
Dietary energy	LE	716.61	897.52	1.33^B^	1,209	1,915	1.58	1,936
	NE	759.68	853.99	1.19^A^	1,191	1,894	1.56	1,978
NPP^1^	LP	569.64	680.63	1.28^b^	1,087	1,653	1.52^A^	1,665
	NP	815.83	968.17	1.25^a^	1,253	2,022	1.60^B^	2,083
	HP	828.97	978.46	1.24^a^	1,260	2,039	1.59^B^	2,125
*p*-value								
Dietary energy		<0.001	0.005	<0.001	0.448	0.566	0.174	0.210
NPP^1^		<0.001	<0.001	0.05	<0.001	<0.001	0.002	<0.001
Interaction		<0.001	0.002	0.287	0.025	0.021	0.482	0.004

^a-c^Within a column, values not sharing a common superscript letter are significantly different at p < 0.05.

^A-C^Within a column, values not sharing a common superscript letter are significantly different at p < 0.01.

^1^NPP: non-phytate phosphorus.

^2^BWG: body weight gain.

^3^FI: feed intake.

^4^FCR: feed conversion ratio.

^5^SEM: standard error of the mean.

*The significance of the main effects cannot be reported when there is significant interactions.

During days of 22–35, weight gain and feed intake were significantly affected by dietary NPP (p < 0.01) and their interaction (p < 0.05). In the MP and HP groups, no difference in BWG or FI was evident at different energy levels. However, in the LP groups, the LE-LP diet showed a significantly greater BWG and higher FI. FCR was only affected by NPP levels (p < 0.01), and LP significantly decreased FCR.

LP significantly decreased the final body weight (p < 0.01) but was not affected by dietary energy. NE-HP showed the highest final body weight.

#### Morphology of the small intestine

In the early growth period, the VH of the duodenum was significantly affected by dietary energy level (p < 0.01) but not by dietary NPP or interaction among energy and NPP levels (Table 4). The VH was significantly smaller in the LE group than in the NE group. CD and VH:CD ratio were significantly affected by dietary energy and their interaction (p < 0.01). LE significantly decreased the CD in all the NPP groups but increased the VH/CD ratio, particularly in the high NPP group.

**Table 4:** The influence of different dietary energy and NPP levels on duodenum morphology in broilers (n = 6).

		21 days			35 days		
Dietary energy	NPP^1^	VH, μm^2^	CD, μm^3^	VH:CD	VH, μm	CD, μm	VH:CD ratio
**Interaction effects**							
LE	LP	1,548	116^A^^B^	13.40^A^	2,170	258.30^a^	8.64^b^
	NP	1,534	128^B^^C^	11.51^A^	1,938	278.88^a^^b^	6.78^a^
	HP	1,529	91^A^	16.92^B^	2,009	305.35^a^^b^	6.60^a^
NE	LP	1,808	148^C^^D^	11.69^A^	2,147	330.37^b^	6.60^a^
	NP	2,068	172^D^^E^	12.10^A^	1,981	280.17^a^^b^	7.08^a^
	HP	2,052	189^E^	10.76^A^	2,044	330.78^b^	6.29^a^
SEM^4^		25.35	2.82	0.28	24.91	5.25	0.156
Main effects*							
Dietary energy	LE	1,537^A^	112	13.94	2,039	280.84	7.34
	NE	1,976^B^	170	11.52	2,058	313.78	6.66
NPP^1^	LP	1,678	132	12.55	2,158^B^	294.34	7.62
	NP	1,801	150	11.8	1,960^A^	279.53	6.93
	HP	1,790	140	13.84	2,027^A^^B^	318.07	6.44
*p*-value							
Dietary energy		<0.001	<0.001	0.003	0.716	0.003	0.035
NPP		0.278	0.13	0.094	0.008	0.016	0.013
Interaction		0.194	0.001	0.003	0.838	0.028	0.011

^a-b^Within a column, values not sharing a common superscript letter are significantly different at p < 0.05.

^A-E^Within a column, values not sharing a common superscript letter are significantly different at p < 0.01.

^1^NPP: non-phytate phosphorus.

^2^VH: villus height.

^3^CD: crypt depth.

^4^SEM: standard error of the mean.

*The significance of the main effects cannot be reported when there is significant interactions.

In the later growth phase, VH was only significantly affected by NPP levels (p < 0.01). LP significantly increased the VH of the intestine. The CD and VH:CD ratio were significantly affected by dietary energy, NPP, and their interaction (p < 0.05). LE-LP significantly decreased the CD of the intestine and enhanced the VH:CD ratio; no difference was found in the other groups.

### Early energy and phosphorus restriction on intestinal phosphate transport and the possible underlying mechanism

#### Adenylate purine concentrations in the duodenum

The low NPP group had significantly lower concentration of AMP and TAN but higher AEC than those in the medium and high NPP groups (p < 0.05; Table 5). The high NPP group had significantly lower concentration of ATP than that in the low and medium NPP groups (p < 0.05). The AMP:ATP ratio was significantly affected by dietary energy, NPP levels, and their interaction (p < 0.05). LE significantly decreased the AMP:ATP ratio, particularly in the MP and HP groups.

**Table 5:** The effect of different dietary energy and NPP levels on adenylate purines in the duodenal mucosa of broilers (n = 6).

Dietary energy	NPP^1^	AMP, μg/g wet weight	ADP, μg/g wet weight	ATP, μg/g wet weight	AMP:ATP	TAN, μg/g wet weight^2^	AEC^3^
**Interaction effects**							
LE	LP	581.13	110.31	124.2	4.68^a^	706.71	0.105
	MP	687.55	148.81	116.3	5.91^a^	876.38	0.091
	HP	633.1	120.81	81.6	7.76^a^	764.29	0.089
NE	LP	554.91	129.21	103.6	5.36^a^	694.48	0.110
	MP	707.02	140	83.6	8.46^b^	829.86	0.091
	HP	681.84	143.1	43.2	15.78^c^	815.11	0.088
SEM^4^		13.03	3.36	5.28	0.55	14.01	0.002
Main effects*							
Dietary energy	LE	640.37	130.55	108.0^b^	5.93	782.46	0.095
	NE	647.92	135.17	74.6^a^	8.69	779.82	0.096
NPP^1^	LP	566.18^A^	121.32	114.9^B^	4.93	700.59^A^	0.107^B^
	MP	708.79^B^	147.09	99.9^B^	7.09	853.12^B^	0.091^A^
	HP	657.47^B^	128.56	59.1^A^	11.12	786.13^B^	0.088^A^
p-value							
Dietary energy		0.811	0.584	0.024	<0.001	0.882	0.909
NPP^1^		0.002	0.053	0.01	<0.001	0.002	0.007
Interaction		0.644	0.284	0.814	0.011	0.558	0.836

^a-c^Within a column, values not sharing a common superscript letter are significantly different at p < 0.05.

^A-B^Within a column, values not sharing a common superscript letter are significantly different at p < 0.01.

^1^NPP: non-phytate phosphorus.

^2^TAN: total adenine nucleotide.

^3^AEC: adenylate energy charge.

^4^SEM: standard error of the mean.

*The significance of the main effects cannot be reported when there is significant interactions.

#### Phosphate transporter expression in the duodenum

**Fig 1:**
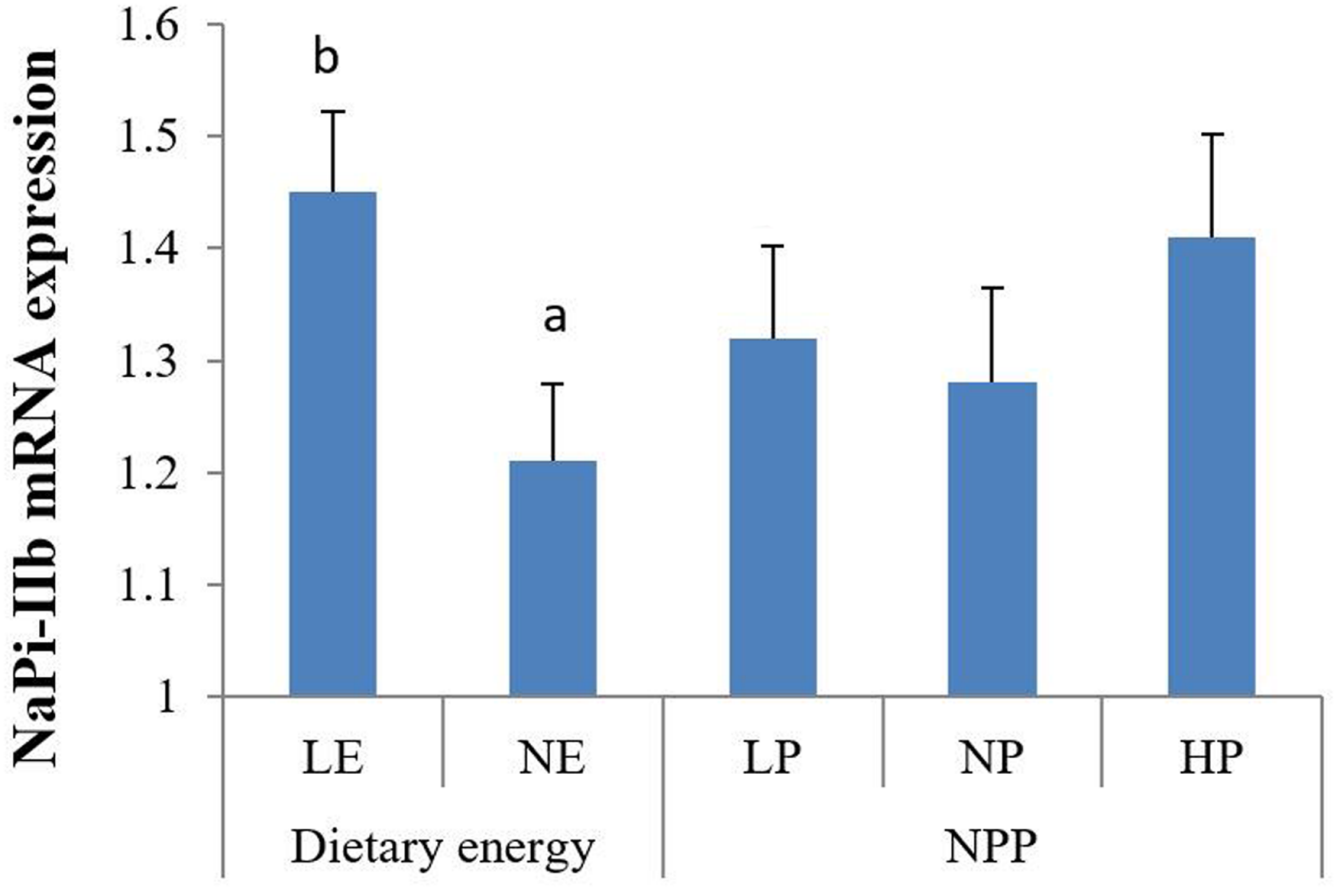
The main effect of dietary energy and NPP levels on phosphorus transporter mRNA expression. Values are means, with standard errors represented by vertical bars. a,b: Mean values within the different dietary energy levels having unlike letters were significantly different (p < 0.05).

NaP_i_-IIb mRNA expression in the duodenum was significantly affected by dietary energy (p < 0.05) but not by NPP levels or their interaction (Table 6, Fig 1). The LE group had significantly higher NaP_i_-IIb mRNA expression than that in the NE group.

**Table 6:** The influence of different dietary energy and NPP levels on phosphorus transporter mRNA expression and AMPK and mTOR phosphorylation in the duodenum of broilers (n = 6).

Dietary energy	NPP^1^	NaP_i_-IIb mRNA expression^2^	p-AMPK:total AMPK ratio	p-mTOR:total mTOR ratio
**Interaction effects**				
LE	LP	1.4	0.98	0.638
	MP	1.48	1.01	0.531
	HP	1.47	0.87	0.572
NE	LP	1.23	0.97	0.393
	MP	1.08	1.07	0.347
	HP	1.33	1.08	0.361
SEM^3^		0.05	0.01	0.012
Main effects*				
Dietary energy	LE	1.45^b^	0.90^a^	0.580^B^
	NE	1.21^a^	1.04^b^	0.367^A^
NPP	LP	1.32	0.98	0.515
	MP	1.28	1.04	0.439
	HP	1.41	0.97	0.466
*p*-values				
Dietary energy		0.022	0.050	<0.001
NPP		0.589	0.397	0.070
Interaction		0.503	0.146	0.607

^a-b^Within a column, values not sharing a common superscript letter are significantly different at p < 0.05.

^A-B^Within a column, values not sharing a common superscript letter are significantly different at p < 0.01.

^1^NPP: non-phytate phosphorus.

^2^NaP_i_-IIb: type IIb sodium-phosphate cotransporter.

^3^SEM: standard error of the mean.

*The significance of the main effects cannot be reported when there is significant interaction.

#### Phosphorylation of AMPK and mTOR

**Fig 2:**
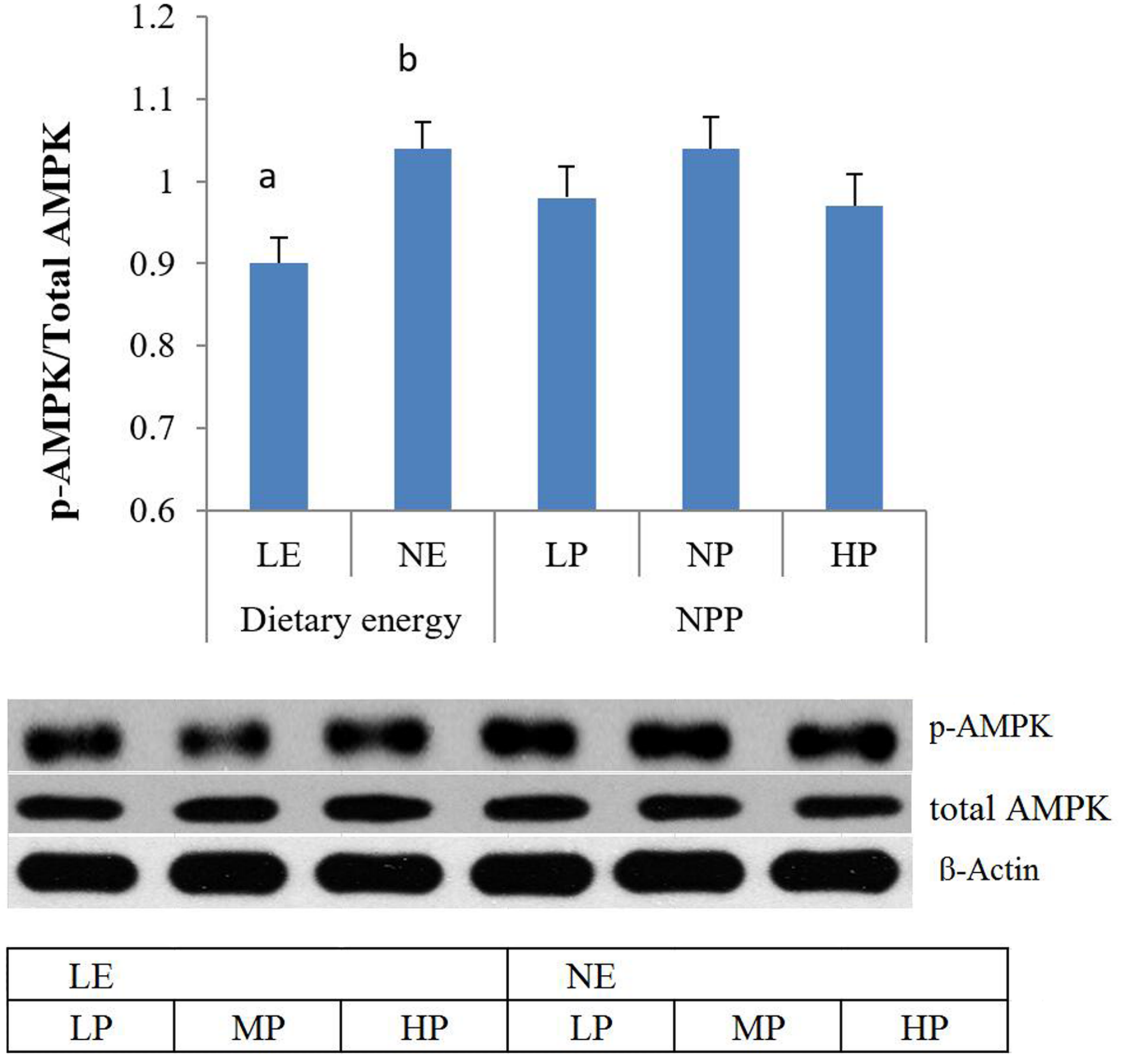
The main effect of dietary energy and NPP levels on the p-AMPK:total AMPK ratio in the intestine. The intensity image is arranged according to the interactions. Values are means, with standard errors represented by vertical bars. a,b: Mean values within the different dietary energy levels having unlike letters were significantly different (p < 0.05).

**Fig 3:**
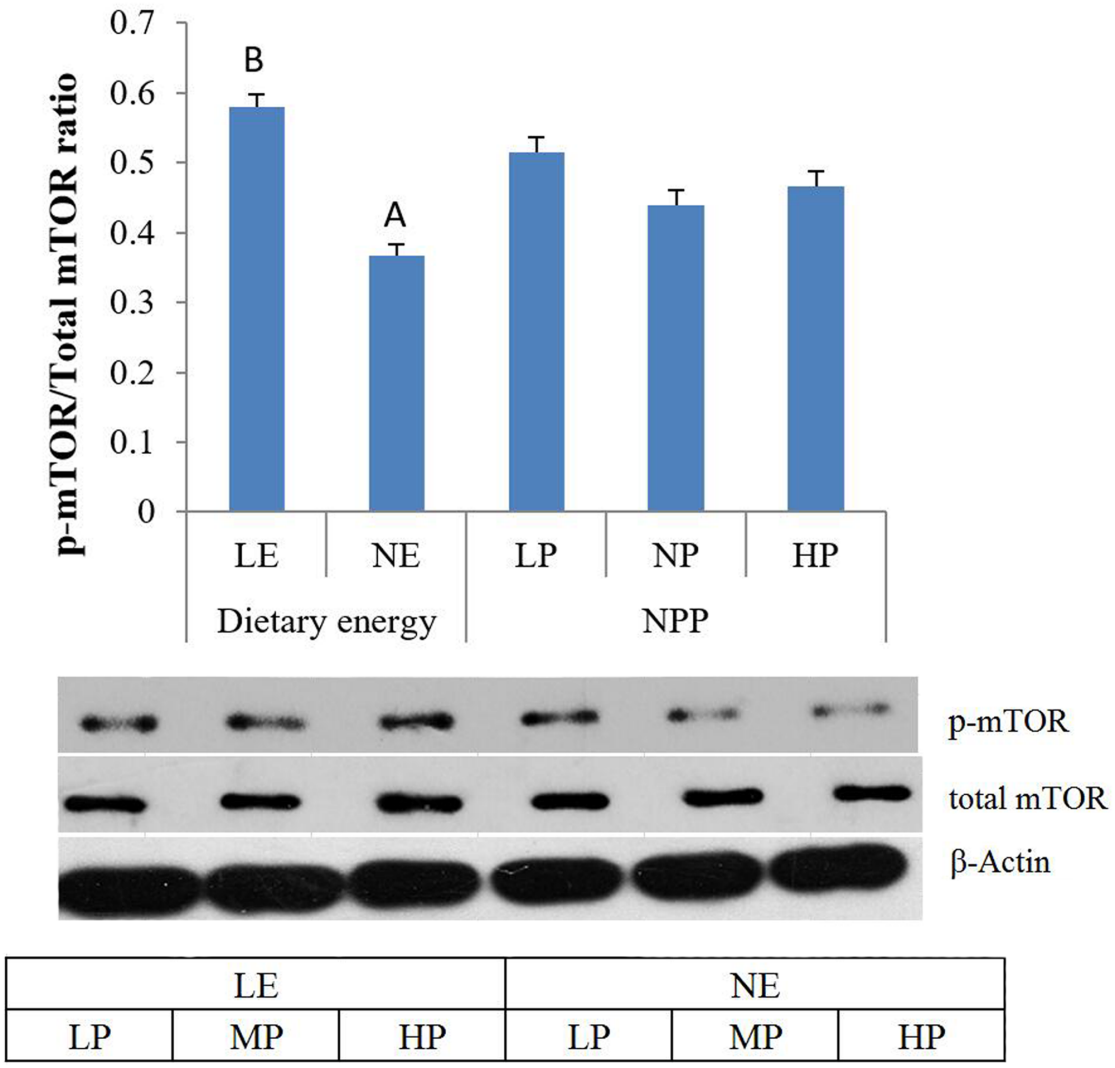
The main effect of dietary energy and NPP levels on the p-mTOR:total mTOR ratio in the intestine. The intensity image is arranged according to the interactions. Values are means, with standard errors represented by vertical bars. A,B: Mean values within the different dietary energy levels having unlike letters were significantly different (p < 0.01).

The levels of phosphorylation of AMPK and mTOR in the duodenum of broilers were determined by western blot. As shown in Fig 2, the ratio of p-AMPK: total AMPK was decreased by low dietary energy (p < 0.05). However, the ratio of p-mTOR: total mTOR was increased by low dietary energy (Fig 3, p < 0.01). The levels of phosphorylation of AMPK and mTOR were not affected by NPP levels and their interaction.

## Discussion

### Compensatory effect of low energy and phosphorus in the early diet

Dietary energy is a dominant factor affecting the growth rate [34], and phosphorus is an important element in body composition [35]. In the present study, LE significantly decreased the early BWG, with no difference in the FI of broilers fed with MP and HP diets; however, this effect was counteracted by the lack of difference in BWG and final body weight of broilers from the age of 22–35 days. In the LP groups, LE increased the FI of broilers, but no difference in BWG was evident compared with NE in the early period. However, broilers on the LE-LP diet showed a significantly greater BWG and FI during days 22–35 and greater final body weight on day 35 compared to broilers on the NE diet. In an earlier study, dietary P deficiency was shown to suppress appetite and growth [9], whereas a low-energy diet was associated with high agouti-related protein mRNA expression in the hypothalamus, which enhanced feed intake [36]. Therefore, LE increased the FI, particularly in the LP group, irrespective of the growth period. Several studies on the effect of dietary energy on BWG have shown that compensatory growth occurs after early energy restriction treatment, thereby enabling the animals to attain normal weights [12, 37]. Chen *et al*. [6] also reported that BWG and feed efficiency during the later growth stages was greater in animals subjected to early energy restriction than those in animals not subjected to this treatment. A possible reason underlying this observation might be that long-term feed restriction reduced the weight maintenance requirements [38, 39] and enhanced nutrient uptake by promoting the development of the digestive system [15, 16]. Furthermore, a study on quail reported that, after cessation of food restriction, the development of some birds in the early period might accelerate during a period of rapid “catch-up” growth, in an attempt to reach an appropriate weight at adulthood [40].

FCE in broilers was related with the functioning of intestinal mitochondria. Intestinal mitochondria in broilers with high FCE exhibited poorer ability to synthesize ATP than did the intestinal mitochondria of broilers with low FCE [25]. In the present study, LP increased the FCR in early stages, i.e., with low FCE. Therefore, an LP diet might have the ability to synthesize much more ATP in intestinal mitochondria, which we will discuss later in this paper. This might explain why the FCR of LP-fed broilers decreased, and FCE was enhanced. Another reason might be that LP could enhance the utilization of nutrients, such as phytate phosphorus, calcium, and phosphorus [41]. In addition, the activity of the type IIb Na-dependent phosphate transporter, which is involved in the regulation of both intestinal and renal reabsorption of inorganic phosphate, could be enhanced throughout the small intestine by LP [22, 42, 43].

Earlier studies reported that although the growth performance of broilers fed with LE was poor, the higher absorption function of the intestine elicits better compensatory growth [15, 16]. Similar to those studies, LE decreased the VH, and the LE-HP group demonstrated the lowest CD and highest VH:CD ratio of the intestine in the early period. A reduction in CD may reduce intestinal maintenance requirement, thereby reserving stores of energy and protein for use in muscle deposition [44]. These results suggest that the absorption function of broilers in the LE group is superior to the secretion function, which is expected to improve the capacity for digestion and absorption of nutrients from the intestine [45, 46]. The early LP exhibited improvement in subsequent intestinal development with higher VH of the intestine, and LE-LP significantly decreased the CD and enhanced the VH:CD ratio. This might have resulted from nutritional programming [16]. The higher VH:CD ratio was consistent with the higher FCE in the LP diet. Intestinal morphology and integrity are related with intestinal energy status [47]. In the present study, this might be ascribed to the difference in the ability of mitochondria to synthesize ATP [25], which warrants further investigation.

### Intestinal phosphate transport and the possible underlying mechanism of early energy and phosphorus restriction

The structure of the small intestine is related to tight junction proteins [48], which control the transport of molecules and act as the intestinal epithelial barrier [49]. Chronic energy restriction has been found to increase the intestinal transport of glucose, fructose, and proline in mice [38]. However, the effect of energy restriction on phosphorus transport has rarely been studied. We found that the LE diet caused significantly higher NaP_i_-IIb mRNA expression than did the NE diet. This finding corresponds with the increase in the VH:CD ratio owing to the LE diet. However, this was inconsistent with a study that showed that long-term caloric restriction markedly reduced the expression of NaP_i_-IIb [24]. This difference might be because of the use of aged mice in that study; here, we selected young, growing birds. The Na-dependent absorption of Pi by the small intestinal brush-border membrane has been suggested to vary with age [50, 51].

In the present study, the effect of dietary energy on FCE might be related to duodenal energy metabolism. The energy charge of the adenyl pool is a better measure of the energy status in tissues than that of a single nucleotide level [32]. The intestine requires a high level of ATP to maintain its integrity, function, and health, and ATP serves as the primary energy source of the cells [52]. AMP is a good indicator of cellular stress, which could accumulate owing to the increased rate of ATP hydrolysis [53]. The novel and important findings of the present study are that the LE diet resulted in increased ATP and a decreased AMP:ATP ratio in the duodenum of chicks. In addition, LP resulted in higher ATP and AEC, lower AMP and AMP:ATP ratio, and decreased TAN in the duodenum of chicks. This result coincides well with the discussion on FCE, and can further explain the effect of LE and LP on the FCE of broilers. LE and LP might reduce the requirement for intestinal maintenance, leading to the accumulation of synthesized ATP in the mitochondria [25, 44].

AMPK, which is a sensor of energy status that maintains cellular energy homeostasis, can be modulated by stimuli that affect cellular ATP levels [54]. It can be phosphorylated and activated by an increase in the AMP:ATP ratio [26]. In the present study, a higher p-AMPK:total AMPK ratio was found in the NE group, which was consistent with a higher AMP:ATP ratio than that in the LE group. Moreover, NaP_i_-IIb in the small intestine might be inhibited by AMPK activation [28], and NaP_i_-IIb gene expression is linked to the metabolic state of the cell through AMPK activity. The potential mechanisms in the regulation of NaP_i_-IIb may involve ADP:ATP or NAD:NADH ratios, because a precursor of NAD has been reported to inhibit intestinal P_i_ absorption [18]. Furthermore, mTOR, which is a kinase known to regulate many intestinal nutrient transporters, is inhibited by the activation of AMPK [55]. NaP_i_-IIb has been shown to be stimulated directly by mTOR [56]. As demonstrated by our study, the relative LE had weaker AMPK phosphorylation, with lower activity to inhibit mTOR phosphorylation. Therefore, the relatively higher mTOR activity would stimulate NaP_i_-IIb mRNA expression in the LE group.

In summary, although low dietary energy reduced the growth performance of broilers in the initial phase, it was beneficial for intestinal development and increased phosphate transport, possibly via intestinal energy metabolism and AMPK signaling. This might explain the compensatory growth induced by low dietary energy. Furthermore, an interaction effect existed between energy and NPP levels on the growth performance and intestinal structure of young broilers. Our findings provide a theoretical basis of intestinal phosphate transport regulation. The mitochondrial functions affected by dietary energy demonstrate a new perspective on intestinal phosphate transport, with potential applications in broiler production.
