# Correction: Effect of early dietary energy restriction and phosphorus level on subsequent growth performance, intestinal phosphate transport, and AMPK activity in young broilers

**DOI:** 10.1371/journal.pone.0192793

**Published:** 2018-02-07

**Authors:** The PLOS ONE Staff

The authors are listed out of order. Please view the correct author order, affiliations, and citation here:

Zhiqiang Miao^1^, Guixian Zhang^1^, Junzhen Zhang^1^, Yu Yang^1^, Jianhui Li^1^

1 College of Animal Science and Veterinary Medicine, Shanxi Agricultural University, Shanxi, China

Miao Z, Zhang G, Zhang J, Yang Y, Li J (2017) Effect of early dietary energy restriction and phosphorus level on subsequent growth performance, intestinal phosphate transport, and AMPK activity in young broilers. PLoS ONE 12(12): e0186828. https://doi.org/10.1371/journal.pone.0186828

The corresponding authors are associated with the following email addresses:

Jianhui Li: jianhui19840717@163.com

Yu Yang: sxauywd@126.com

The publisher apologizes for this error.

## References

[1] Miao Z, Zhang G, Zhang J, Li J, Yang Y (2017) Effect of early dietary energy restriction and phosphorus level on subsequent growth performance, intestinal phosphate transport, and AMPK activity in young broilers. PLoS ONE 12(12): e0186828 https://doi.org/10.1371/journal.pone.0186828 29240752 10.1371/journal.pone.0186828 PMC5730151

